# A dataset of 1050-tampered color and grayscale images (CG-1050)

**DOI:** 10.1016/j.dib.2019.104864

**Published:** 2019-11-21

**Authors:** Maikol Castro, Dora M. Ballesteros, Diego Renza

**Affiliations:** Universidad Militar Nueva Granada, Colombia

**Keywords:** Fake image, Tampering detection, Forgery detection, Copy-move, Cut-paste, Retouching, Colorizing

## Abstract

This paper presents the CG-1050 dataset consisting of 100 original images, 1050 tampered images and their corresponding masks. The dataset is organized into four directories: *original* images, *tampered* images, *mask* images, and a *description* file. The directory of original images includes 15 color and 85 grayscale images. The directory of tampered images has 1050 images obtained through one of the following type of tampering: *copy-move*, *cut-paste*, *retouching*, and *colorizing*. The true mask between every pair of original and its tampered image is included in the mask directory (1380 masks). The description file shows the names of the images (i.e., original, tampered and mask), the image description, the photo location, the type of tampering, and the manipulated object in the image. With this dataset, the researchers can train and validate fake image classification methods, either for labelling the tampered image or for forgery pixel-detection.

**Table:** Specifications Table

Subject	Computer Vision and Pattern Recognition
Specific subject area	Image processing related to identify/classify tampered data
Type of data	Images Table
How data were acquired	The original images were captured with the camera Huawei ref. ANE-LX3 in the following places: street (51 photos), park (20 photos), touristic place (11), mall (8 photos), shop (4 photos), classroom (2 photos), parking lot (1 photo), room (1 photo), kitchen (1 photo), and playroom (1 photo). The tampered images were obtained by using Adobe Photoshop.
Data format	Raw: original images (JPEG) Modified (copy-move, cut-paste, retouching, colorizing): tampered images (JPEG) Mask images (PNG)
Parameters for data collection	Four types of tampering were performed: copy-move, cut-paste, retouching and colorizing
Description of data collection	The dataset is composed by four directories, organized as follows: 1 Original: 100 images (i.e., 15 color and 85 grayscale images) 2 Tampered: 1050 images (i.e., 338 images with copy-move operation, 50 images of cut-paste forgery, 308 retouched images and 354 colorized images) 3 Mask: 100 sub-directories with the corresponding masks between the pairs of original and tampered images 4 Description: a spreadsheet file with the description of the dataset content
Data source location	City: Bogotá Country: Colombia
Data accessibility	Repository name: Mendeley Data name: CG-1050: Original and tampered images (Color and grayscale) [1] Direct URL to data: https://doi.org/10.17632/dk84bmnyw9.2

**Table:** 

**Value of the Data** • All the original images are real photos captured in different indoor/outdoor places. The tampered images are created using Adobe Photoshop, providing a natural effect not obvious to the human eye. The modified pixels correspond to realistic regions instead of fixed blocks. • Most of the tampered image datasets available for benchmarking are focused on only one or two types of tampering [2], for example, the IMD and the MICC-F600 for copy-move operation, or the CASIA v2.0 dataset for copy–move and cut–paste manipulations. Unlike the above, our dataset includes the following manipulations: copy–move, cut–paste, retouching and colorizing. This allows training and validating image tampering detection models for a wider scenario. • Some of the tampered image datasets available for benchmarking do not include the true mask [2], like the Columbia gray, the CASIA, and the MICC-F2000. In our dataset, for every tampered image, the true mask is provided; in addition, for color images, there is a mask for every color band. This allows evaluating the accuracy of forgery-pixel detection methods. • The ratio between the number of tampered images and the number of and original images is 10/1, being higher than the ratio in other datasets such as COVERAGE (1/1), MICC-F600 (4/11), and CASIA v2.0 (5/7). This characteristic is useful to avoid overfitting, as the model is trained with several examples of tampered images by each original image.

## 1 Data

The dataset is organized in four directories: *Original* images, *Tampered* images, *Mask* images, and a *Description* file [1].

**Fig. 1:**
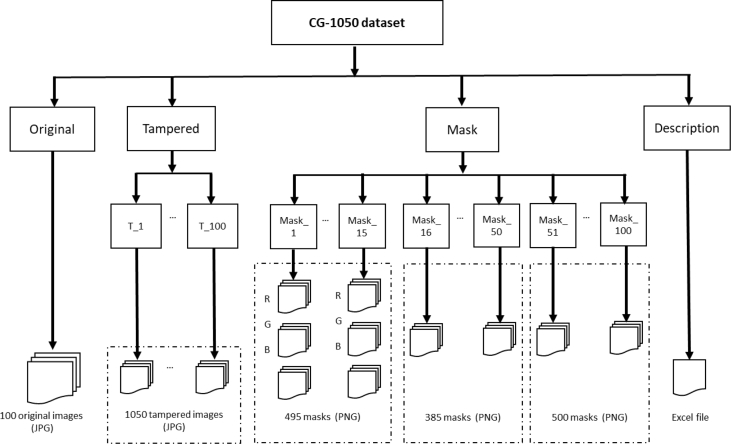
CG-1050 dataset structure.

Fig. 1 shows the structure of the dataset, which is explained below:

• The directory of *Original* images includes 15 color and 85 grayscale images. All original images are in JPEG format.
• The directory of *Tampered* images has 100 sub-directories (i.e., T_1 to T_100) with 1050 images obtained through one of the following types of tampering: *copy-move*, *cut-paste*, *retouching*, and *colorizing.* The first 50 sub-directories have 11 tampered images, each one; the last 50 sub-directories have 10 tampered images by directory. All tampered images are in JPEG format.
• The directory named *Mask* has 100 sub-directories (i.e., Mask_1 to Mask_100) with their true masks obtained by each pair of original and tampered image. In the case of color images, every of the 15 sub-directories has 11 folders and 3 masks by folder, that is, 495 masks. In the case of grayscale images, every one of the first 35 sub-directories have 11 masks; and every one of the last 50 sub-directories have 10 masks, i.e., 885 masks. To sum up, the entire CG-1050 dataset has 1380 masks. In the mask image, the manipulated pixels are black and the unmodified pixels are white.
• Finally, the directory named *Description* has an excel file with information about the dataset details: original images (i.e., photo name, image description, photo place), tampered images (i.e., folder name, type of tampering, tampered photo name, object, location), and mask (i.e., folder name, mask photo name).

**Fig. 2:**
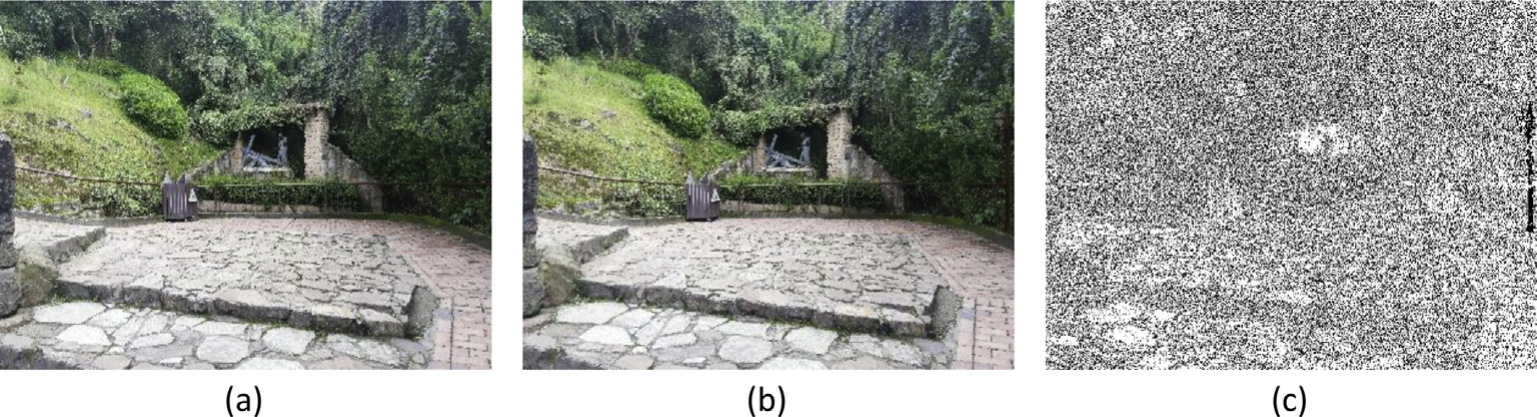
An example of cut-paste manipulation: a) original image, b) tampered image, c) mask (green band).

**Fig. 3:**
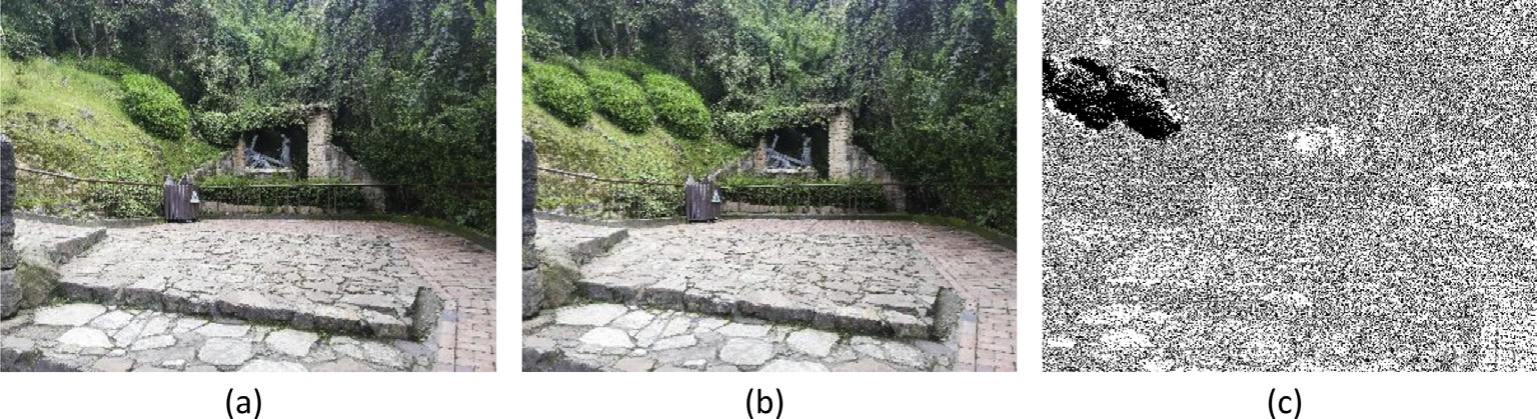
An example of copy-move manipulation: a) original image, b) tampered image, c) mask.

**Fig. 4:**
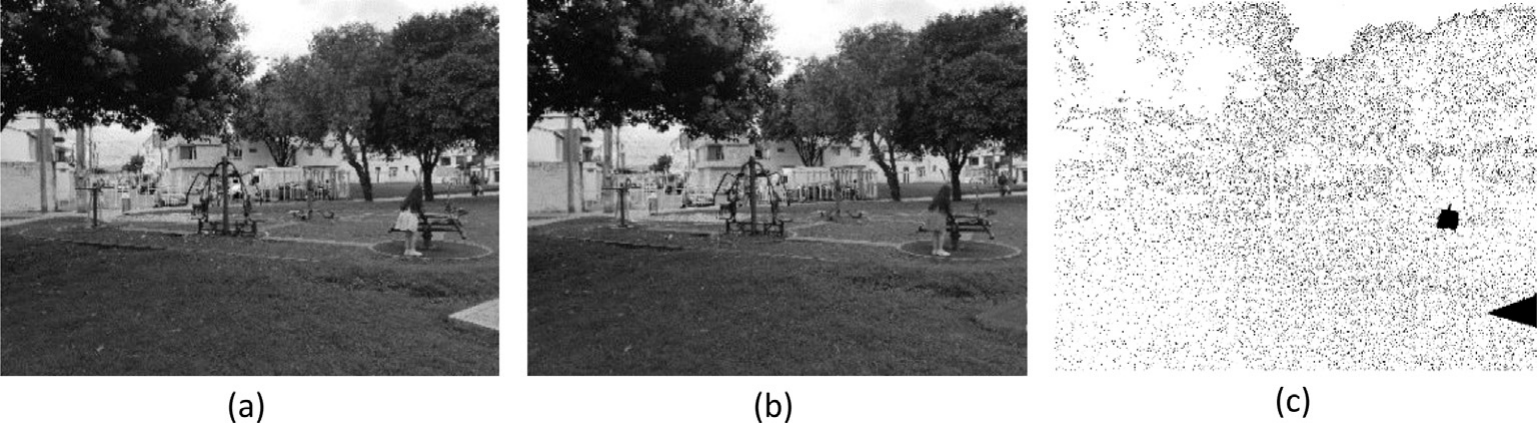
An example of colorizing: a) original image, b) tampered image, c) mask.

**Fig. 5:**
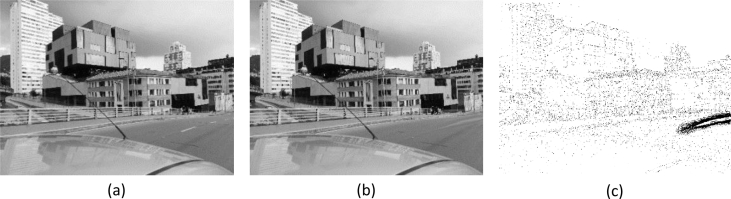
An example of retouching: a) original image, b) tampered image, c) mask.

Fig. 2 shows an example of cut-paste manipulation for a color image, with the original, the tampered image, and the mask. Fig. 3 shows an example of a copy-move operation and associated images. Fig. 4, Fig. 5 show an example of grayscale images for colorizing and retouching. Table 1, Table 2, Table 3, Table 4 describe the information in Fig. 2, Fig. 3, Fig. 4.

**Table 1:** Data description of the cut-paste example.

Original image	Photo name	Im_9
	Image description	Color 4608 × 3456
	Photo place	Touristic place
Tampered image	Folder name	T_9
	Type of Tampering	Cut-paste
	Photo name (tampered)	Im9_cmfr1.jpg
	Object	Light pole
	Location	Middle right
Mask image	Folder name	Mask_9
	Photo name (mask)	Mask8_cmfr1.png

**Table 2:** Data description of the copy-move example.

Original image	Photo name	Im_9
	Image description	Color 4608 × 3456
	Photo place	Touristic place
Tampered image	Folder name	T_9
	Type of Tampering	Copy-move
	Photo name (tampered)	Im9_cm1.jpg
	Object	Bush
	Location	Upper left
Mask image	Folder name	Mask_9
	Photo name (mask)	Mask9_cm1.png

**Table 3:** Data description of the colorizing example.

Original image	Photo name	Im_29
	Image description	Grayscale 4608 × 3456
	Photo place	Park
Tampered image	Folder name	T_29
	Type of Tampering	Colorizing
	Photo name (tampered)	Im29_col2.jpg
	Object	Sidewalk and a dress
	Location	Low right
Mask image	Folder name	Mask_29
	Photo name (mask)	Mask29_col2.png

**Table 4:** Data description of the retouching example.

Original image	Photo name	Im_80
	Image description	Grayscale 4608 × 3456
	Photo place	Street
Tampered image	Folder name	T_80
	Type of Tampering	Retouching
	Photo name (tampered)	Im80_r3.jpg
	Object	Lines of the road
	Location	Middle right
Mask image	Folder name	Mask_80
	Photo name (mask)	Mask80_r3.png

## 2 Experimental design, materials, and methods

The natural images were captured in the following places: street (51 photos), park (20 photos), touristic place (11), mall (8 photos), shop (4 photos), classroom (2 photos), parking lot (1 photo), room (1 photo), kitchen (1 photo), and playroom (1 photo). Size of the images are (3456 × 4608) or (4608 × 3456) pixels. For every original image, 10 to 11 tampered images (i.e., with copy-move [3,4], cut-paste [2], retouching [5,6] and colorizing [7,8]) are obtained.

Fig. 2 shows an example of *cut-paste* modification of a color image. The left plot is the original image, the middle plot is the tampered image, and the right plot is its corresponding mask (G band). The light pole located in the right side of Fig. 2b is the object copied from another image. Table 1 shows the details of these images found in the *Description* directory.

Fig. 3 shows another example of tampered color images. In this case, a *copy-move* modification is applied to the original image, pasting twice a bush. The true mask is presented in Fig. 3c. Table 2 shows the details of the original, tampered and mask images for this example.

For the third example, a grayscale image of the CG-1050 dataset is selected. The intensity of the girl's dress is changed as well as the intensity of the sidewalk. Fig. 4 shows the original image, the tampered image and its mask. Table 3 lists the details of these images.

The last example is shown in Fig. 5. The lines of the road are blurred, through a retouching effect. The tampered object is located in the middle right of the image (see Fig. 5c). Table 4 shows the details of this manipulation.
